# Structural Insights into the Cytotoxic Mechanism of *Vibrio parahaemolyticus* PirA*^vp^* and PirB*^vp^* Toxins

**DOI:** 10.3390/md15120373

**Published:** 2017-12-01

**Authors:** Shin-Jen Lin, Kai-Cheng Hsu, Hao-Ching Wang

**Affiliations:** 1 Institute of Bioinformatics and Biosignal Transduction, College of Bioscience and Biotechnology, National Cheng Kung University, Tainan 701, Taiwan; f90225004@ntu.edu.tw; 2 Graduate Institute of Cancer Biology and Drug Discovery, College of Medical Science and Technology, Taipei Medical University, Taipei 110, Taiwan; piki@tmu.edu.tw; 3 Graduate Institute of Translational Medicine, College of Medical Science and Technology, Taipei Medical University, Taipei 110, Taiwan

**Keywords:** shrimp disease, AHPND, *Photorhabdus* insect-related toxin, PirA*^vp^*, PirB*^vp^*

## Abstract

In aquaculture, shrimp farming is a popular field. The benefits of shrimp farming include a relatively short grow-out time, high sale price, and good cost recovery. However, outbreaks of serious diseases inflict serious losses, and acute hepatopancreatic necrosis disease (AHPND) is an emerging challenge to this industry. In South American white shrimp (*Penaeus vannamei*) and grass shrimp (*Penaeus monodon*), this disease has a 70–100% mortality. The pathogenic agent of AHPND is a specific strain of *Vibrio parahaemolyticus* which contains PirA*^vp^* and PirB*^vp^* toxins encoded in the pVA1 plasmid. PirA*^vp^* and PirB*^vp^* have been shown to cause the typical histological symptoms of AHPND in infected shrimps, and in this review, we will focus on our structural understanding of these toxins. By analyzing their structures, a possible cytotoxic mechanism, as well as strategies for anti-AHPND drug design, is proposed.

## 1. Introduction

Acute hepatopancreatic necrosis disease (AHPND), which was originally known as early mortality syndrome (EMS), first broke out in China in 2009, then spreading to Vietnam, Malaysia and Thailand [1,2]. Because of this disease, shrimp production dropped to ~60% compared with 2012, and total economic losses have been estimated at more than $1 billion per year, globally [3]. The causative agent of AHPND was soon found to be a specific strain of *Vibrio parahaemolyticus*. *V. parahaemolyticus* is a halophilic Gram-negative bacterium that is commonly found in estuarine, marine and coastal environments [4], and originally it was not known how this opportunistic bacterium had become virulent and capable of causing disease in shrimps.

In addition to the readily observable symptoms in infected *P. monodon* and *P. vannamei*—lethargy, an empty stomach and midgut, and a pale to white atrophied hepatopancreas [4]—histological examination of the diseased shrimp further showed that the HP tubule epithelial cells sloughed into the HP tubule lumens [4,5]. Meanwhile, in the initial, acute stage of AHPND, even when a large number of bacteria could be found in the stomach, there were still sometimes no obvious bacterial colonies in the hepatopancreas tube lumens [1,4,6]. This led Tran et al. to propose that the symptoms of AHPND were caused by a toxin secreted by the pathogen [4]. This proposal was further supported by reverse gavage experiments in which introduction of the bacteria-free supernatant of the bacterial culture into healthy shrimp induced typical AHPND symptoms [4,7]. 

Subsequent investigations focused on isolating AHPND variants [8] and on comparing the draft genome sequences of AHPND-causing versus non-AHPND-causing strains [1,9,10,11,12]. By using a next-generation sequencing (NGS) platform to sequence and compare three virulent (3HP, 5HP and China) and one non-virulent (S02) *V. parahaemolyticus* strains [9], Yang et al. (2014) found that a 69-kb extrachromosomal plasmid was present in all AHPND-causing strains but not in the non-virulent strain. This plasmid was named pVA1, and sequence analysis showed that it contained homologs of the insecticidal *Photorhabdus* insect-related (Pir) binary toxin PirA/PirB [13]. The importance of these two toxins to AHPND was confirmed by subsequent studies [14,15,16], and they are now referred to as *V. parahaemolyticus* PirA/PirB (PirA*^vp^*/PirB*^vp^*).

## 2. The Structural Similarity between *V. parahaemolyticus* PirA*^vp^*/PirB*^vp^* and *Bacillus thuringiensis* Cry Toxins

*Photorhabdus* PirA and PirB were first reported as potential toxins by genomic sequencing of the entomopathogenic bacterium *Photorhabdus luminescens* W14 [17], and in 2009, Waterfield et al. reported that both *Photorhabdus* PirA and PirB were necessary for the insecticidal activity against caterpillars of the moth *Galleria mellonella* [18]. Although sequence similarity had previously led to *Photorhabdus* PirB being initially identified as a juvenile hormone esterase-like (JHE-like) protein [19], Waterfield et al. found that *Photorhabdus* PirB did not have JHE activity [20], and another study further showed that it had sequence similarity to the pore-forming domain I of the *B. thuringiensis* Cry toxin [21]. However, although it was established that *Photorhabdus* PirA/PirB was an effective insecticidal binary toxin [18,20,21,22], its cytotoxic mechanism remained unclear. 

The first crystal structures to be reported for any PirA/PirB toxins were for *V. parahaemolyticus* PirA*^vp^* and PirB*^vp^*, and the accompanying structural analysis also suggested a relationship between *B. thuringiensis* Cry and PirA*^vp^*/PirB*^vp^* toxins [13]. Cry proteins are one of the *B. thuringiensis* insecticidal toxins, and they have an important potential use in agriculture [23,24]. Although Cry toxins can be divided into at least 75 primary subgroups, and can show differences in their amino acid sequences, the determined and predicted structures of almost all of the Cry toxins are similar [25]. Cry toxins have three functional domains: the pore-forming domain I, the receptor-binding domain II and the sugar-binding domain III [23,24,25,26,27,28,29]. The specificity and cytotoxic mechanisms of Cry toxins are mediated by these three domains, and they have been discussed in many review articles [23,24,25,28,29,30,31]. For example, *B. thuringiensis* Cry1A uses domains II and III to target receptors that are abundant in the midgut of insect larvae, such as alkaline phosphatase (ALP) or aminopeptidase N (APN). The concentrated Cry1A toxins then interact with another receptor, cadherin-like receptor (CAD), which facilitates the proteolytic cleavage of its domain I helix α1. This cleavage induces the formation of the Cry oligomer, which uses the activated domain I to form non-selective pores in the apical membrane. This causes colloidal osmotic lysis of the cells.

**Figure 1:**
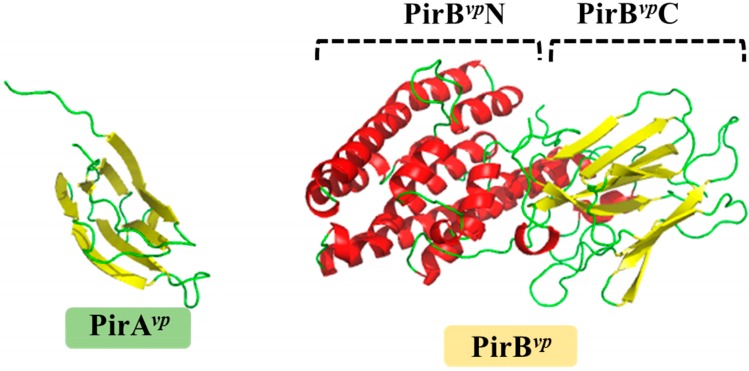
Crystal structures of PirA*^vp^* (**left**) and PirB*^vp^* (**right**) toxins. The α-helices and β-strands are shown in red and yellow, respectively. PirA*^vp^* has a jelly-roll topology which is folded into an eight-stranded antiparallel β-barrel. PirB*^vp^* has two domains with distinct structural features: the N-terminal of PirB*^vp^* (PirB*^vp^*N; residues 12–256) forms a seven-α-helix bundle; while the C-terminal (PirB*^vp^*C; residues 279–436) contains two pairs of four-stranded antiparallel β-sheets. PirB*^vp^*N and PirB*^vp^*C are connected by a long loop. The PDB codes 3X0T and 3X0U were used to produce the figures for PirA*^vp^* and PirB*^vp^*, respectively.

**Figure 2:**
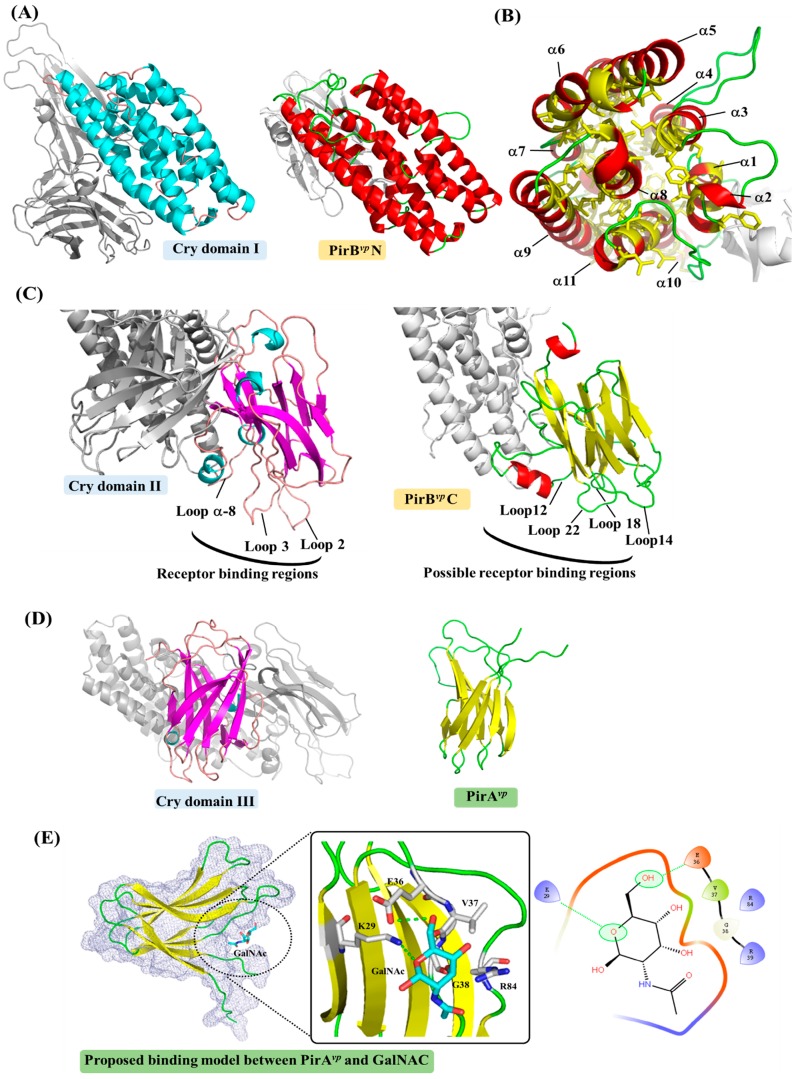
A detailed comparison between structures of *B. thuringiensis* Cry and PirA*^vp^*/PirB*^vp^* toxins. The α-helices and â-sheets of Cry domain I and PirA*^vp^*/PirB*^vp^* are colored cyan and magenta, and red and yellow, respectively. (**A**) A comparison between Cry domain I and PirB*^vp^*N; (**B**) Inside the α-helical bundle of PirB*^vp^*N. The hydrophobic residues Leu, Ile, Val, Met, Phe, Trp and Cys are shown in yellow; (**C**) A comparison between Cry domain II and PirB*^vp^*C showing the receptor binding loops of Cry domain II. A possible receptor-binding region of PirB*^vp^*C is proposed based on a structural comparison to Cry domain II; (**D**) A comparison between Cry domain III and PirA*^vp^*; (**E**) A potential ligand-binding site of PirA*^vp^*. GalNAc is shown docked into the structure of PirA*^vp^* using the docking tool iGEMDOCK [35]. Briefly, each atom of the residues and the compound was first assigned an atom type (e.g., donor or acceptor) and formal charge based on their physiochemical properties. The scoring function of iGEMDOCK was then used to measure intermolecular interactions between PirA*^vp^* and GalNAc. In this docking model, the oxygen heteroatom of GalNAc forms hydrogen bonds with residue Lys29. Residue Glu36 yields a hydrogen bond with one of GalNAc’s hydroxyl groups. Gly38 is a non-polar residue that is sandwiched in close proximity to two hydroxyl groups. Residues Val37 and Arg84 interact with the compound via van der Waals forces. The PDB code 1CIY was used to produce the figures for the Cry toxin.

Figure 1 shows the crystal structure of the PirA*^vp^* and PirB*^vp^* toxins. Figure 2 shows how PirB*^vp^* corresponds to Cry domains I and II, while PirA*^vp^* has similar topology to Cry domain III. These structural similarities suggest PirA*^vp^*/PirB*^vp^* binary toxin is a Cry-like, three-domain toxin, but with a dissociated domain III [13,27]. The following sections discuss this idea in more detail.

### 2.1. PirB^vp^ Contains Both Cry-Like Pore-Forming and Receptor Domains

Both the N-terminal domain of PirB*^vp^* (PirB*^vp^*N) and Cry domain I contain a bundle of α-helices (Figure 2A) [13]. Figure 2B further shows that there are abundant hydrophobic residues located in the center of the PirB*^vp^*N α-bundle, and that the hydrophobic α-helix 8 of PirB*^vp^*N is sheltered within a bundle of amphipathic α-helices. This “inside-out membrane fold” is consistent with other pore-forming toxins that can switch between soluble and transmembrane conformations [32]. After triggering conformational change, these hydrophobic residues become exposed on the surface of the protein, where they are able to interact with membrane lipids. It should also be noted that these helices are generally longer than 40 Å, which is sufficient to cross the cell membrane (the length of the lipid bilayer is ~40 Å). Similar features are seen in the pore-forming domain I of Cry toxin, as well as in other pore-forming toxins, like colicin [28,29,32,33]. All of this strongly suggests that PirB*^vp^*N has the ability to form a pore on the cell membrane that causes cell death. Meanwhile, the C-terminal domain of PirB*^vp^* (PirB*^vp^*C) has three antiparallel β-sheets arranged in a manner similar to that seen in Cry domain II (Figure 2C) [13]. Since the Cry domain II contains an immunoglobulin-like folding that is involved in protein–protein or protein–ligand interactions [34], it seems likely that the PirB*^vp^*C domain plays a similar functional role. Further, since Cry domain II could interact with insect receptors [23,24,25,26,29,30,31], the structural similarity suggests PirB*^vp^*C is also a receptor binding domain.

### 2.2. PirA^vp^ Contains a Possible Sugar-Binding Pocket

Like PirB*^vp^*, the biological functions of PirA*^vp^* may also be revealed by its structural features. PirA*^vp^* contains two antiparallel β-sheets that are packed together in a jelly-roll topology [13]. This folding is similar to domain III of the Cry toxin (Figure 2D). Cry domain III contains a galactose-binding domain-like fold [36,37]; this is thought to be related to the toxin’s specificity via its recognition of receptor-bound *N*-acetylgalactosamine (GalNAc) [23,24,25,26,29,30,31,36,37,38]. In the interaction between Cry1Ac and APN, Cry1Ac domain III first interacts with the GalNAc sugar on the APN receptor to facilitate the subsequent toxin-receptor binding [23]. PirA*^vp^* does indeed play a similar role to Cry domain III, then it should facilitate target-specific recognition by binding to certain ligands on the cell membrane/receptor. Interestingly, a potential sugar-binding cavity formed by three loops was found in PirA*^vp^* (Figure 2E). The docking model shows that when the GalNAc molecule was fitted into this cavity, it could potentially interact with the PirA*^vp^* residues Lys29, Glu36, Val37, Gly38 and Arg84 (Figure 2E). We further note that, since the potential binding cavity of PirA*^vp^* is deep and narrow (Figure 2E), it may be possible that PirA*^vp^* not only targets the monosaccharides like GalNAc, but also oligosaccharides.

### 2.3. Unanswered Questions Relating to the Cytotoxic Mechanism of PirA^vp^/PirB^vp^

We have shown that the PirA*^vp^*/PirB*^vp^* toxin has structures that are similar to the functional domains of Cry. This further suggests that PirA*^vp^*/PirB*^vp^* might also induce cell death via the respective Cry-like steps of receptor binding, oligomerization and pore forming. To explore this model, identification of the cell receptors that might interact with PirA*^vp^*/PirB*^vp^* is a logical place to start. We note that although the main folding of Cry domain II and PirB*^vp^*C is similar, the loop regions between these two domains are quite different (Figure 2C). Since the loop á-8, loop 2 and loop 3 of Cry Domain II are very important to aminopeptidase N (APN)-, alkaline phosphatase (ALP)- and cadherin (CAD)-receptor binding [26,39,40,41], these divergent loop regions suggest either that the toxin-interacting regions on shrimp’s APN, ALP and CAD receptors are different to those found in insects, or else that PirB*^vp^* targets different receptors on the shrimp cell’s membrane. In either case, given that PirA*^vp^*/PirB*^vp^* toxin induces cell death in the shrimp’s hepatopancreas, but not in the stomach or other organs, it seems very likely that these putative PirA*^vp^*/PirB*^vp^* receptors will be found exclusively in the hepatopancreas membrane. However, we caution that there is as yet no experimental evidence in support of this; at present, the structure of these shrimp receptors remains unknown. We also note that several other critical processes still need to be investigated experimentally. For example, we do not yet know whether the cleavage of N-terminal á-helices on PirB*^vp^* is important for toxin activation, or whether PirA*^vp^*/PirB*^vp^* forms an oligomer in order to make a pore in the membrane.

Determination of the binding ligand of PirA*^vp^* is also worth investigating. Although the binding model between PirA*^vp^* and GalNAc seems reasonable, this interaction still needs to be confirmed by experiments such as surface plasmon resonance. To explore more possibilities, a high-throughput screening of PirA*^vp^* bound ligands would be useful, and we note that a feasible chip platform designed for carbohydrate-protein interactions has recently been developed [42,43,44].

**Figure 3:**
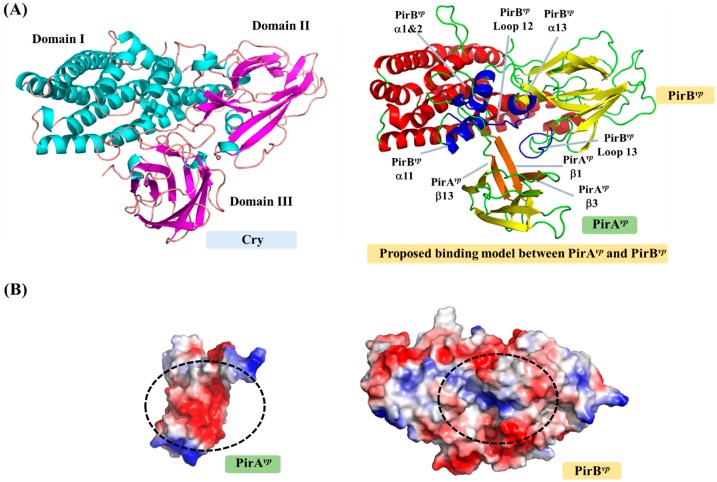
Possible binding mode and interface between PirA*^vp^* and PirB*^vp^* toxins. (**A**) Cry and proposed PirA*^vp^*/PirB*^vp^* complex. The PirA*^vp^*/PirB*^vp^* complex was predicted by reference to the positions of the three Cry domains. The possible binding regions of PirA*^vp^* and PirB*^vp^* are colored orange and blue; (**B**) The surface charges on the complex interfaces of PirA*^vp^* and PirB*^vp^*. Red and blue respectively indicate negatively and positively charged regions.

To become a true three-domain toxin, PirA*^vp^* and PirB*^vp^* must first form a complex. Although the complex formation of PirA*^vp^*/PirB*^vp^* was confirmed using gel filtration [13], the resulting structure is still unknown, so how these two toxins bind to each other is still unclear. Based on the locations of the corresponding domains in the Cry toxin, a possible binding model of PirA*^vp^* and PirB*^vp^* was proposed (Figure 3; [13]). In this model, á-helices 1, 2, 12 and 13, and loops 12 and 13 of PirB*^vp^* create a potential binding cavity for PirA*^vp^*, while the â-sheets 1, 3 and 9 of PirA*^vp^* interact with PirB*^vp^* (Figure 3A). Figure 3B shows how the surface charges on the PirA*^vp^*/PirB*^vp^* interface are complementary to each other, further suggesting that this model is reasonable. However, as noted above, this PirA*^vp^*/PirB*^vp^* binding model still needs to be verified experimentally.

Furthermore, although there are many structural similarities, some physiological characteristics between Cry and PirA*^vp^*/PirB*^vp^* toxins may be different. For example, the Cry protoxins generally form crystals in the mother cell compartment [45,46]. Since the crystals have to be solubilized in the gut of insect larvae to become biologically active, this ability of the protoxins to crystallize may decrease their susceptibility to premature proteolytic degradation [45]. Previous reports have shown that the solubility of these Cry crystals is dependent on pH [45,47,48]; the crystals that form in the neutral pH of the mother cells subsequently dissolve in the acidic environment (<pH 4) of the insect gut. However, unlike Cry toxins, there are no reports of in vivo crystal formation for PirA*^vp^*/PirB*^vp^*, and although in vivo crystallization of Cry toxins is an important control step of their toxicities, it seems unlikely that PirA*^vp^*/PirB*^vp^* would use a similar control mechanism. Nevertheless this has not yet been demonstrated experimentally.

A more complete understanding of the cytotoxic mechanisms of PirA*^vp^*/PirB*^vp^* toxins is likely to be important for AHPND research, but could also be important for agricultural applications. Although there is genetic distance between PirA*^vp^*/PirB*^vp^* and the PirA/PirB homologs that are found in other bacteria such as *Photorhabdus asymbiotica* (WP_015835800/WP_015835799) [18], *Photorhabdus luminescens* (ABE68878/ABE68879) [19], *Xenorhabdus doucetiae* (CDG18638/CDG18639), *Xenorhabdus cabanillasii* (CDL79383/CDL79384), *Xenorhabdus nematophila* (WP013183676/WP010845483) and *Alcaligenes faecalis* (WP003801867/WP003801865), these insecticidal PirA and PirB toxins have allowed *Photorhabdus* and *Xenorhabdus* to be used in biological pest control [18,21,22]. The study of PirA*^vp^*/PirB*^vp^* should also therefore provide useful information for insecticidal applications.

## 3. Strategies for Designing Drugs to Block the Cytotoxic Effects of *V. parahaemolyticus* PirA*^vp^* and PirB*^vp^* Toxins

Although AHPND-detection methods that can monitor the shrimps and the environment during cultivation have already been developed [7,49,50], there are still no available drugs that can be used in the treatment of AHPND. It has already been clearly established that PirA*^vp^* and PirB*^vp^* toxins are the main cytotoxic source of AHPND; for example, the deletion/mutation of their *pirA^vp^* and *pirB^vp^* genes from pVA1 can decrease AHPND severity and reduce the mortality of the shrimps [12,13,15,16]. Additionally, PirA*^vp^* and PirB*^vp^* are both secreted proteins [13,15], which means that they could be easily targeted by drugs/inhibitors. Neutralization of PirA*^vp^* and/or PirB*^vp^* toxicity is therefore a rational direction for AHPND drug design. Further, since the structures of PirA*^vp^* and PirB*^vp^* are both available, a structure-based drug design can be used to achieve this goal more efficiently. 

**Figure 4:**
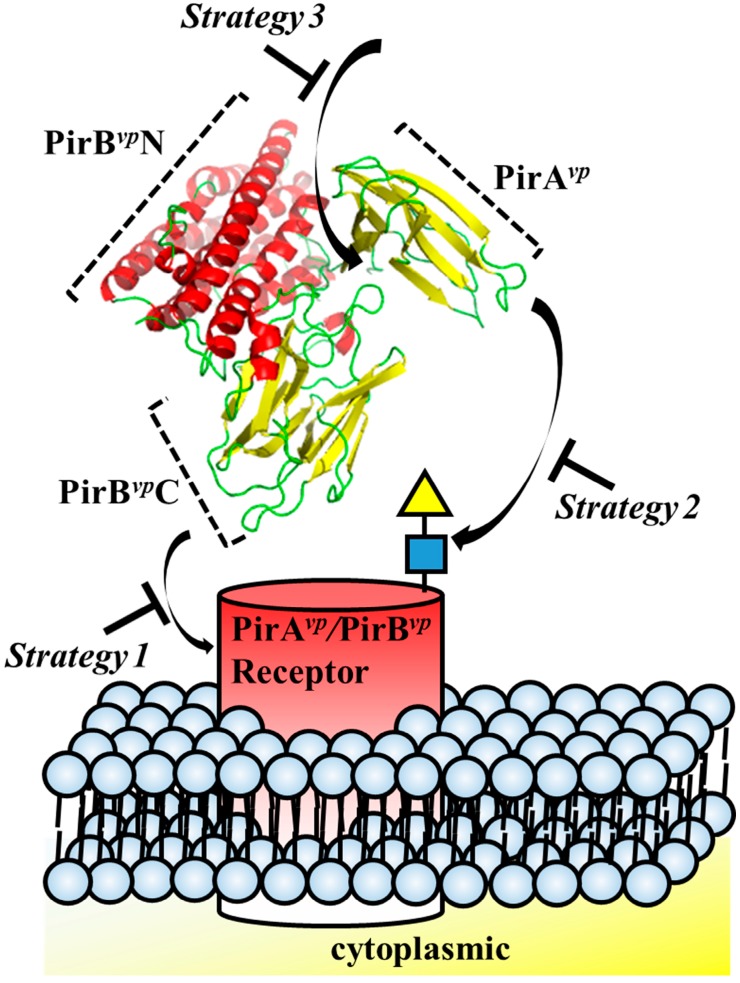
Strategies for designing drugs to block the cytotoxic effects of PirA*^vp^* and PirB*^vp^* toxins.

Structure-based drug design has been successfully used before. For example, in the well-studied pore-forming toxins, such as colicin and hemolysin, structural biology provided a wealth of useful knowledge regarding conformation rearrangement, receptor/ligand binding regions and oligomerization [32]. Structural insights into toxins also enables the development of novel therapeutic strategies [32]. For example, small molecules or engineered antibodies can be designed to interact with specific sites on the toxins. In the case of *Aeromonas hydrophila* aerolysin, which targets glycosylphosphatidylinisotol (GPI)-anchored proteins, synthetic GPI molecules and GPI analogues have been proposed as inhibitors [51]. It has also recently been shown that *Staphylococcus aureus* hemolysin can be neutralized by an antibody that targets the receptor binding site of this toxin [52]. Similarly, with other pore-forming toxins, receptors that bind these toxins, such as CCR5 and ADAM10, can also be considered in a reverse strategy for drug design [53,54]. For example, Leukotoxin ED pore-forming toxin targets human CCR5 receptor, and CCR5 receptor antagonists such as maraviroc were shown to block Leukotoxin ED-induced cell death [53]. The structural characteristics of PirA*^vp^* and PirB*^vp^* suggests three regions that are potentially suitable for structure-based drug design: (1) the potential receptor-binding region of PirB*^vp^*; (2) ligand-binding region of PirA*^vp^* and (3) the interacting region between PirA*^vp^* and PirB*^vp^* (Figure 4, Table 1). Interface information such as amino acid sequences and structural motifs can be used for antibody engineering, as well as for in silico compound screening.

Although engineered antibodies can be used to investigate the importance of these various PirA*^vp^* and PirB*^vp^* regions in the laboratory, they are probably too expensive and difficult to use in the field. By contrast, small compounds may be more suitable for AHPND treatment in aquaculture. Recently, in silico screening approaches have been used to identify small molecules that disrupt protein–protein interactions [55,56], and currently, data is available for over 35 million compounds on databases such as ZINC (http://zinc.docking.org/; [57]). By using in silico screening approaches, these compounds can be virtually docked into specific sites on PirA*^vp^* or PirB*^vp^*. Furthermore, the binding affinities between PirA*^vp^*/PirB*^vp^* and compounds can be predicted using molecular docking tools, such as iGEMDOCK [35] and AutoDock Vina [58]. Ligand-based screening is another approach that can be used to identify inhibitors [59]. On the assumption that similar compounds can mimic physicochemical properties of the interacting regions and occupy the interface of the target protein, this approach uses online chemistry tools (e.g., Open Babel; [60]) to search for compounds that are similar to interacting peptides (e.g., a loop) of partner proteins (e.g., the PirA*^vp^* binding interface on PirB*^vp^*). Ultimately, compounds with high docking scores that predict greater affinity can be considered as potential inhibitors, and these can then be validated through bioassays, as well as shrimp challenge assays. Using these approaches, we are hopeful that a potential PirA*^vp^*/PirB*^vp^* drug/inhibitor can be discovered in the near future.

## 4. Conclusions

In this review, we have presented structural views of the major pathogenic factors of AHPND: *V. parahaemolyticus* PirA*^vp^* and PirB*^vp^*. Based on the structural similarity to *B. thuringiensis* Cry pore-forming toxin, we hypothesized that PirA*^vp^* and PirB*^vp^* may use similar mechanisms to cause cell death in shrimps. Furthermore, strategies for drug/inhibitor design against these two toxins were proposed. As more details are discovered, we anticipate that the future safety and usefulness of the insecticidal applications of this toxin family will also be improved.

## Figures and Tables

**Table 1:** Potential interacting regions on PirA*^vp^* and PirB*^vp^* that may be suitable targets for structure-based drug design.

Potential Function	Regions Involved in Possible Interactions	Amino Acid Sequences
Receptor binding	PirB*^vp^* Loop 12	274-VGFPS-278
	PirB*^vp^* Loop 14	316-SIEIHYYNRV-325
	PirB*^vp^* Loop 18	369-GPE-371
	PirB*^vp^* Loop 22	413-QEGSDKV-419
PirA*^vp^*/PirB*^vp^* complex formation	PirA*^vp^* β-sheet 1	11-YSHDWTV-17
	PirA*^vp^* β-sheet 3	26-VDSKH-30
	PirA*^vp^* β-sheet 9	104-GFCTIYY-110
	PirB*^vp^* α-helix 1	35-YAFKAMVSFG-43
	PirB*^vp^* α-helix 2	45-LSN-47
	PirB*^vp^* α-helix 12	247-MILWQKIKEL-256
	PirB*^vp^* α-helix 13	260-DVFVHSNLISY-270
	PirB*^vp^* Loop 12	298-PNMFGERR-305
	PirB*^vp^* Loop 13	431-PDEF-434
Ligand binding	PirA*^vp^* β-sheet 3	26-VDS**K**H-30 *
	PirA*^vp^* Loop 4	31-TPIIP**EVG**RS-40 *
	PirA*^vp^* Loop 8	83-Q**R**PDNAFY-90 *

* GalNAc interacting residues are shown in bold.

## References

[1] Gomez-Gil B., Soto-Rodriguez S., Lozano R., Betancourt-Lozano M. (2014). Draft Genome Sequence of *Vibrio parahaemolyticus* Strain M0605, Which Causes Severe Mortalities of Shrimps in Mexico. Genome Announc..

[2] Nunan L., Lightner D., Pantoja C., Gomez-Jimenez S. (2014). Detection of acute hepatopancreatic necrosis disease (AHPND) in Mexico. Dis. Aquat. Org..

[3] FAO Fisheries and Aquaculture Report of the FAO/MARD Technical Workshop on Early Mortality Syndrome (EMS) or Acute Hepatopancreatic Necrosis Syndrome (AHPNS) of Cultured Shrimp (under TCP/VIE/3304). www.fao.org/docrep/018/i3422e/i3422e.pdf.

[4] Tran L., Nunan L., Redman R., Mohney L., Pantoja C., Fitzsimmons K., Lightner D. (2013). Determination of the infectious nature of the agent of acute hepatopancreatic necrosis syndrome affecting penaeid shrimp. Dis. Aquat. Org..

[5] Lightner D.V., Redman R.M., Pantoja C.R., Noble B.L., Tran L. (2012). Early mortality syndrome affects shrimp in Asia. Glob. Aquac. Advocate.

[6] Soto-Rodriguez S.A., Gomez-Gil B., Lozano-Olvera R., Betancourt-Lozano M., Morales-Covarrubias M.S. (2014). Field and Experimental Evidence of *Vibrio parahaemolyticus* as the Causative Agent of Acute Hepatopancreatic Necrosis Disease of Cultured Shrimp (*Litopenaeus vannamei*) in Northwestern Mexico. Appl. Environ. Microbiol..

[7] Sirikharin R., Taengchaiyaphum S., Sanguanrut P., Chi T.D., Mavichak R., Proespraiwong P., Nuangsaeng B., Thitamadee S., Flegel T.W., Sritunyalucksana K. (2015). Characterization and PCR Detection Of Binary, Pir-Like Toxins from *Vibrio parahaemolyticus* Isolates that Cause Acute Hepatopancreatic Necrosis Disease (AHPND) in Shrimp. PLoS ONE.

[8] Joshi J., Srisala J., Truong V.H., Chen I.T., Nuangsaeng B., Suthienkul O., Lo C.F., Flegel T.W., Sritunyalucksana K., Thitamadee S. (2014). Variation in *Vibrio parahaemolyticus* isolates from a single Thai shrimp farm experiencing an outbreak of acute hepatopancreatic necrosis disease (AHPND). Aquaculture.

[9] Yang Y.-T., Chen I.-T., Lee C.-T., Chen C.-Y., Lin S.-S., Hor L.-I., Tseng T.-C., Huang Y.-T., Sritunyalucksana K., Thitamadee S. (2014). Draft Genome Sequences of Four Strains of *Vibrio parahaemolyticus*, Three of Which Cause Early Mortality Syndrome/Acute Hepatopancreatic Necrosis Disease in Shrimp in China and Thailand. Genome Announc..

[10] Kondo H., Tinwongger S., Proespraiwong P., Mavichak R., Unajak S., Nozaki R., Hirono I. (2014). Draft Genome Sequences of Six Strains of *Vibrio parahaemolyticus* Isolated from Early Mortality Syndrome/Acute Hepatopancreatic Necrosis Disease Shrimp in Thailand. Genome Announc..

[11] Gomez-Jimenez S., Noriega-Orozco L., Sotelo-Mundo R.R., Cantu-Robles V.A., Cobian-Guemes A.G., Cota-Verdugo R.G., Gamez-Alejo L.A., Del Pozo-Yauner L., Guevara-Hernandez E., Garcia-Orozco K.D. (2014). High-quality draft genomes of two *Vibrio parahaemolyticus* strains aid in understanding acute hepatopancreatic necrosis disease of cultured shrimps in Mexico. Genome Announc..

[12] Han J., Tang K., Tran L., Lightner D. (2015). Photorhabdus insect-related (Pir) toxin-like genes in a plasmid of *Vibrio parahaemolyticus*, the causative agent of acute hepatopancreatic necrosis disease (AHPND) of shrimp. Dis. Aquat. Org..

[13] Lee C.-T., Chen I.-T., Yang Y.-T., Ko T.-P., Huang Y.-T., Huang J.-Y., Huang M.-F., Lin S.-J., Chen C.-Y., Lin S.-S. (2015). The opportunistic marine pathogen *Vibrio parahaemolyticus* becomes virulent by acquiring a plasmid that expresses a deadly toxin. Proc. Natl. Acad. Sci. USA.

[14] Lai H.-C., Ng T.H., Ando M., Lee C.-T., Chen I.-T., Chuang J.-C., Mavichak R., Chang S.-H., Yeh M.-D., Chiang Y.-A. (2015). Pathogenesis of acute hepatopancreatic necrosis disease (AHPND) in shrimp. Fish Shellfish Immunol..

[15] Tinwongger S., Nochiri Y., Thawonsuwan J., Nozaki R., Kondo H., Awasthi S.P., Hinenoya A., Yamasaki S., Hirono I. (2016). Virulence of acute hepatopancreatic necrosis disease PirAB-like relies on secreted proteins not on gene copy number. J. Appl. Microbiol..

[16] Theethakaew C., Nakamura S., Motooka D., Matsuda S., Kodama T., Chonsin K., Suthienkul O., Iida T. (2017). Plasmid dynamics in *Vibrio parahaemolyticus* strains related to shrimp Acute Hepatopancreatic Necrosis Syndrome (AHPNS). Infect. Genet. Evol..

[17] Ffrench-Constant R.H., Waterfield N., Burland V., Perna N.T., Daborn P.J., Bowen D., Blattner F.R. (2000). A Genomic Sample Sequence of the Entomopathogenic Bacterium Photorhabdus luminescens W14: Potential Implications for Virulence. Appl. Environ. Microbiol..

[18] Ahantarig A., Chantawat N., Waterfield N.R., ffrench-Constant R., Kittayapong P. (2009). PirAB Toxin from *Photorhabdus asymbiotica* as a Larvicide against Dengue Vectors. Appl. Environ. Microbiol..

[19] Duchaud E., Rusniok C., Frangeul L., Buchrieser C., Givaudan A., Taourit S., Bocs S., Boursaux-Eude C., Chandler M., Charles J.F. (2003). The genome sequence of the entomopathogenic bacterium *Photorhabdus luminescens*. Nat. Biotechnol..

[20] Waterfield N., Kamita S.G., Hammock B.D., ffrench-Constant R. (2005). The *Photorhabdus* Pir toxins are similar to a developmentally regulated insect protein but show no juvenile hormone esterase activity. FEMS Microbiol. Lett..

[21] Ffrench-Constant R.H., Dowling A., Waterfield N.R. (2007). Insecticidal toxins from *Photorhabdus* bacteria and their potential use in agriculture. Toxicon.

[22] Li Y., Hu X., Zhang X., Liu Z., Ding X., Xia L., Hu S. (2014). *Photorhabdus luminescens* PirAB-fusion protein exhibits both cytotoxicity and insecticidal activity. FEMS Microbiol. Lett..

[23] Bravo A., Gill S.S., Soberón M. (2007). Mode of action of *Bacillus thuringiensis* Cry and Cyt toxins and their potential for insect control. Toxicon.

[24] Pardo-López L., Soberón M., Bravo A. (2013). *Bacillus thuringiensis* insecticidal three-domain Cry toxins: Mode of action, insect resistance and consequences for crop protection. FEMS Microbiol. Rev..

[25] Adang M., Crickmore N., Jurat-Fuentes J. (2014). Diversity of *Bacillus thuringiensis* Crystal Toxins and Mechanism of Action. Adv. Insect Physiol..

[26] Bravo A., Gómez I., Porta H., García-Gómez B.I., Rodriguez-Almazan C., Pardo L., Soberón M. (2012). Evolution of *Bacillus thuringiensis* Cry toxins insecticidal activity. Microb. Biotechnol..

[27] Berry C., Crickmore N. (2017). Structural classification of insecticidal proteins—Towards an in silico characterisation of novel toxins. J. Invertebr. Pathol..

[28] Li J., Carroll J., Ellar D.J. (1991). Crystal structure of insecticidal δ-endotoxin from *Bacillus thuringiensis* at 2.5 Å resolution. Nature.

[29] Grochulski P., Masson L., Borisova S., Pusztai-Carey M., Schwartz J.L., Brousseau R., Cygler M. (1995). *Bacillus thuringiensis* CryIA(a) insecticidal toxin: Crystal structure and channel formation. J. Mol. Biol..

[30] Soberón M., Pardo L., Muñóz-Garay C., Sánchez J., Gómez I., Porta H., Bravo A. (2010). Pore formation by Cry toxins. Adv. Exp. Med. Biol..

[31] Pigott C.R., Ellar D.J. (2007). Role of receptors in *Bacillus thuringiensis* crystal toxin activity. Microbiol. Mol. Biol. Rev..

[32] Peraro M.D., van der Goot F.G. (2015). Pore-forming toxins: Ancient, but never really out of fashion. Nat. Rev. Microbiol..

[33] Parker M.W., Pattus F., Tucker A.D., Tsernoglou D. (1989). Structure of the membrane-pore-forming fragment of colicin A. Nature.

[34] Williams A.F., Barclay A.N. (1988). The immunoglobulin superfamily—Domains for cell surface recognition. Annu. Rev. Immunol..

[35] Hsu K.C., Chen Y.F., Lin S.R., Yang J.M. (2011). iGEMDOCK: A graphical environment of enhancing GEMDOCK using pharmacological interactions and post-screening analysis. BMC Bioinform..

[36] Kitami M., Kadotani T., Nakanishi K., Atsumi S., Higurashi S., Ishizaka T., Watanabe A., Sato R. (2011). *Bacillus thuringiensis* Cry Toxins Bound Specifically to Various Proteins via Domain III, Which Had a Galactose-Binding Domain-Like Fold. Biosci. Biotechnol. Biochem..

[37] Sengupta A., Sarkar A., Priya P., Ghosh Dastidar S., Das S. (2013). New Insight to Structure-Function Relationship of GalNAc Mediated Primary Interaction between Insecticidal Cry1Ac Toxin and HaALP Receptor of *Helicoverpa armigera*. PLoS ONE.

[38] Jenkins J.L. (2000). Bivalent Sequential Binding Model of a *Bacillus thuringiensis* Toxin to Gypsy Moth Aminopeptidase N Receptor. J. Biol. Chem..

[39] Gomez I., Oltean D.I., Gill S.S., Bravo A., Soberon M. (2001). Mapping the epitope in cadherin-like receptors involved in *Bacillus thuringiensis* Cry1A toxin interaction using phage display. J. Biol. Chem..

[40] Gomez I., Miranda-Rios J., Rudino-Pinera E., Oltean D.I., Gill S.S., Bravo A., Soberon M. (2002). Hydropathic Complementarity Determines Interaction of Epitope (869)HITDTNNK(876) in *Manduca sexta* Bt-R1 Receptor with Loop 2 of Domain II of *Bacillus thuringiensis* Cry1A Toxins. J. Biol. Chem..

[41] Gómez I., Dean D.H., Bravo A., Soberón M. (2003). Molecular Basis for *Bacillus thuringiensis* Cry1Ab Toxin Specificity: Two Structural Determinants in the *Manduca sexta* Bt-R1Receptor Interact with Loops α-8 and 2 in Domain II of Cy1Ab Toxin. Biochemistry.

[42] Park S., Lee M.R., Pyo S.J., Shin I. (2004). Carbohydrate Chips for Studying High-Throughput Carbohydrate−Protein Interactions. J. Am. Chem. Soc..

[43] Seo J.H., Kim C.S., Hwang B.H., Cha H.J. (2010). A functional carbohydrate chip platform for analysis of carbohydrate–protein interaction. Nanotechnology.

[44] Dan X., Liu W., Ng T. (2016). Development and applications of lectins as biological tools in biomedical research. Med. Res. Rev..

[45] Schnepf H.E., Crickmore N., van Rie J., Lereclus D., Baum J., Feitelson J., Zeigler D.R., Dean D.H. (1998). *Bacillus thuringiensis* and its pesticidal crystal proteins. Microbiol. Mol. Biol. Rev..

[46] Adalat R., Saleem F., Crickmore N., Naz S., Shakoori A.R. (2017). In vivo crystallization of three-domain Cry toxins. Toxins.

[47] Koller C.N., Bauer L.S., Hollingworth R.M. (1992). Characterization of the pH-mediated solubility of Bacillus *thuringiensis* var. san diego native δ-endotoxin crystals. Biochem. Biophys. Res. Commun..

[48] Deist B., Rausch M., Fernandez-Luna M., Adang M., Bonning B. (2014). Bt Toxin Modification for Enhanced Efficacy. Toxins.

[49] Flegel T.W., Lo C.F. (2013). Free Release of Primers for Specific Detection of Bacterial Isolates that Cause Acute Hepatopancreatic Necrosis Disease (AHPND).

[50] Dangtip S., Sirikharin R., Sanguanrut P., Thitamadee S., Sritunyalucksana K., Taengchaiyaphum S., Mavichak R., Proespraiwong P., Flegel T.W. (2015). AP4 method for two-tube nested PCR detection of AHPND isolates of *Vibrio parahaemolyticus*. Aquac. Rep..

[51] Wu Q., Guo Z. (2010). Glycosylphosphatidylinositols are potential targets for the development of novel inhibitors for aerolysin-type of pore-forming bacterial toxins. Med. Res. Rev..

[52] Foletti D., Strop P., Shaughnessy L., Hasa-Moreno A., Casas M.G., Russell M., Bee C., Wu S., Pham A., Zeng Z. (2013). Mechanism of Action and In Vivo Efficacy of a Human-Derived Antibody against *Staphylococcus aureus* α-Hemolysin. J. Mol. Biol..

[53] Alonzo F., Kozhaya L., Rawlings S.A., Reyes-Robles T., DuMont A.L., Myszka D.G., Landau N.R., Unutmaz D., Torres V.J. (2012). CCR5 is a receptor for *Staphylococcus aureus* leukotoxin ED. Nature.

[54] Inoshima I., Inoshima N., Wilke G.A., Powers M.E., Frank K.M., Wang Y., Wardenburg J.B. (2011). A *Staphylococcus aureus* pore-forming toxin subverts the activity of ADAM10 to cause lethal infection in mice. Nat. Med..

[55] Sliwoski G., Kothiwale S., Meiler J., Lowe E.W. (2014). Computational methods in drug discovery. Pharmacol. Rev..

[56] Zhong S.J., Macias A.T., MacKerell A.D. (2007). Computational identification of inhibitors of protein-protein interactions. Curr. Top. Med. Chem..

[57] Irwin J.J., Shoichet B.K. (2005). ZINC—A Free Database of Commercially Available Compounds for Virtual Screening. J. Chem. Inf. Model..

[58] Trott O., Olson A.J. (2010). Software News and Update AutoDock Vina: Improving the speed and accuracy of docking with a new scoring function, efficient optimization, and multithreading. J. Comput. Chem..

[59] Eckert H., Bajorath J. (2007). Molecular similarity analysis in virtual screening: Foundations, limitations and novel approaches. Drug Discov. Today.

[60] O’Boyle N.M., Banck M., James C.A., Morley C., Vandermeersch T., Hutchison G.R. (2011). Open Babel: An open chemical toolbox. J. Cheminform..

